# Association between breastfeeding and breast, thyroid, and cervical cancer among Korean adult women based on the Korean Genome and Epidemiology Study: a cohort study

**DOI:** 10.4069/kjwhn.2021.11.29

**Published:** 2021-12-13

**Authors:** Eunju Jin, Hyunju Kang, Mia Son

**Affiliations:** 1 Department of Nursing, Graduate School, Kangwon National University, Chuncheon, Korea; 2 College of Nursing, Kangwon National University, Chuncheon, Korea; 3 College of Medicine, Kangwon National University, Chuncheon, Korea

**Keywords:** Breast feeding, Breast neoplasms, Thyroid neoplasms, Uterine cervical neoplasms

## Abstract

**Purpose:**

The purpose of this study was to explore the association between breastfeeding and the prevalence of breast, thyroid, and cervical cancer among Korean adult women.

**Methods:**

The study was a secondary analysis of data from the Korea Genome and Epidemiology Study. The final samples were 113,944 Korean women among 173,205 urban-based cohort participants collected between 2004 and 2013 for adults aged forty and over. To determine the association between female cancers and breastfeeding experience, the number of childbirth, and total breastfeeding duration, logistic regression analysis was done. The demographic characteristics, health behavior, and female history were adjusted.

**Results:**

The prevalence of breast cancer was 1.37 times higher in the non-breastfeeding group than in the breastfeeding group. Compared to having breastfed for more than 36 months, the prevalence of thyroid cancer was 1.68 times higher at breastfeeding for 13 to 36 months, 1.67 times higher at breastfeeding for 6 to 12 months, and 2.06 times higher at breastfeeding less than 6 months. Also, the prevalence of cervical cancer was 1.54 times higher at breastfeeding for 13 to 36 months, compared to breastfeeding for more than 36 months.

**Conclusion:**

This study found that breastfeeding experience and a longer breastfeeding duration are associated with reduced risk of breast, thyroid, and cervical cancer in Korean women. It can be used as a basis for encouraging breastfeeding, and suggests further research on modifiable factors that reduce cancer risks.

## Introduction

Cancer is the leading cause of death in Korea, and the number of new cancer patients in 2018 was 235,547, a year-on-year increase of 3.5% [1]. If we assume 86 years as an average Korean woman’s life expectancy, one in three (34.2%) women are estimated to develop cancer during their lifetime [2]. There are sex-specific differences in the incidence and mortality associated with some cancers; cervical cancer, in particular, develops only in women, while both breast and thyroid cancer have been reported to occur more often in women than men [1-3].

In Korea, the prevalence of breast cancer has steadily increased over the past 20 years. According to the National Cancer Registry, breast cancer ranked first among cancers diagnosed in women in 2018. Previous studies have demonstrated dose-response relationships between tobacco and alcohol consumption and breast cancer risk, and an increased risk of breast cancer has been reported in postmenopausal women with higher body mass index (BMI) and in cases where physical activity is insufficient [4-6]. Moreover, cumulative exposure to sex hormones—such as estrogen and progesterone—acts as an important mechanism toward developing key risk factors for breast cancer. Factors contributing to cumulative exposure to sex hormones—namely age, early menarche, late menopause, and old-age childbirth—have been reported to be associated with breast cancer risk [4]. Thyroid cancer is the second most common cancer among women, next only to breast cancer [1]. Thyroid cancer is one of the cancer types with a high 5-year survival rate [2], but a relatively high recurrence rate and a poor prognosis when it metastasizes to other sites in the body [7]. Older age at menopause was found to be related to a weakly increased risk of thyroid cancer, and a longer duration of breastfeeding was associated with a moderately reduced risk of thyroid cancer [8]. The prevalence of cervical cancer has decreased since the introduction of national cancer screening in Korea, but cervical cancer is still reported at a rate that is six times higher than the mean prevalence of developed nations, such as the United States and Europe [9]. The human papillomavirus (HPV) has been identified as a high-risk factor for cervical cancer. Other factors related to cervical cancer have included early sexual experiences, unsafe sex life, consuming oral contraceptives, poverty, smoking, nutrition, and immunosuppression state [9,10].

Breastfeeding benefits not only the infant but also the mother. During motherhood, breastfeeding helps reduce postpartum weight gain by consuming calories, releases the hormone oxytocin, helps shrink the size of the uterus back to the pre-pregnancy state, and reduces uterine bleeding [11,12]. In addition to these immediate effects after childbirth, the long-term maternal health benefits of breastfeeding include reduced risks of osteoporosis, metabolic disease, and rheumatoid arthritis [13]. Previous studies have particularly reported on the relationship between breastfeeding and lowered cancer risk in women [8,14,15]. In a systematic literature review and meta-analysis, Chowdhury et al. [14] reported that compared with mothers who did not breastfeed, mothers who breastfed for more than 12 months had a 26% lower risk of developing breast cancer. According to a meta-analysis, late menopause and old-age childbirth increased the risk of thyroid cancer, whereas a long breastfeeding duration contributed to preventing thyroid cancer [8]. In the study conducted by Yi et al. [15], breastfeeding and the risk of thyroid cancer were found to be inversely correlated, and the risk of thyroid cancer decreased with an increase in the duration of breastfeeding. Relative risk for an increment of 1 month of breastfeeding with risk of thyroid cancer was 0.983 [15].

In the case of Korean women, a trend emerged where decreasing breast cancer risk was associated with an increased duration of breastfeeding [16,17], while the total duration of breastfeeding significantly decreased the risk of thyroid cancer [18]. However, it is hard to find a study that confirms the link between breastfeeding and cervical cancer. In addition, it is necessary to identify and compare how breastfeeding duration is associated with breast cancer, thyroid cancer, and cervical cancer. Additionally, in this simultaneous comparison, it is necessary to comprehensively control and analyze variables including sociodemographic characteristics, health behaviors, and various aspects of reproductive history that can affect cancer incidence.

This study aims to determine the association between breastfeeding and the prevalence of thyroid cancer and cervical cancer, which are representative female cancers, in an urban cohort collected from the Korean Genome and Epidemiology Study (KoGES), conducted by the National Institute of Health (NIH), and the Korea Centers for Disease Control and Prevention (KCDC)—renamed the Korea Disease Control and Prevention Agency in September 2020. The KoGES urban cohort is a large-scale cohort constituting adults aged 40 years and over, who have visited the medical screening center in urban areas since 2004 to identify risk factors for major chronic diseases among Koreans [19]. In particular, this is intended to determine the epidemiologic association between breastfeeding-related variables and breast cancer, thyroid cancer, and cervical cancer prevalence rates after adjustment for demographic characteristics (e.g., age, education level, and socioeconomic status), health behavioral characteristics (e.g., smoking, drinking, and exercise), and reproductive history variables (e.g., menarche and menopause) simultaneously. Doing so, the methodology of the study is aimed at verifying and comparing the effects of breastfeeding on the occurrence of major female cancers through the recent representative cohort data.

The purpose of this study was to determine the association between breastfeeding and the prevalence of breast, thyroid, and cervical cancers, which are representative female cancers, among Korean adult women aged 40 years and over. The specific aims were as follows: first, to check the differences in the prevalence of breast, thyroid, and cervical cancers depending on sociodemographic characteristics, health behaviors, and reproductive history; and second, to determine the effects of breastfeeding experience, number of childbirths, and total breastfeeding duration on the prevalence of breast, thyroid, and cervical cancers.

## Methods

Ethics statement: This study was exempted by the Institutional Review Board of Kangwon National University (KWNUIRB-2021-02-009) as this was secondary analysis of existing data and the data were anonymous.

### Study design

This is a secondary data analysis study using data collected from the report of KoGES (KoGES: 4851-302), conducted by the NIH/KCDC. This study was designed according to the STROBE (Strengthening the Reporting of Observational Studies in Epidemiology) guidelines for reporting cohort studies, available from: https://www.strobe-statement.org/.

### Setting/data sources

The KoGES is a cohort project carried out to establish a scientific framework for personalized preventive medicine, based on epidemiological research, by identifying the risk factors of frequent chronic diseases among Koreans [19]. Among the KoGES cohorts, the urban-based cohort consisted of adult males and females over the age of 40 years who visited medical examination centers in 14 cities across the country. The survey period was from 2004 to 2013, and participants were recruited and surveyed by each performing institution. The contents of the survey were questionnaire items, including general matters, disease history, disease treatment status, drug history, family history, and lifestyle habits such as drinking, smoking, and physical activity. The examination items included recording blood pressure, pulse measurement, body composition analysis, clinical examination, electrocardiogram, radiographic examination, and physical measurement.

### Participants

Of the total 173,205 participants, 113,944 were female: all female participants were the final analysis target in this study.

### Study variables

*Breastfeeding experience*: Breastfeeding experience was classified by answering “Yes” or “No” to the question, “Have you ever fed your own milk to your baby?”

*Number of childbirths*: The number of childbirths was classified into one, two, and three or more.

*Total breastfeeding duration*: The total duration of breastfeeding was classified as under 6 months, 6 to 12 months, 13 to 36 months, and 36 months or more.

*Prevalence of female cancers*: For the purpose of this study, the operational definition of female cancers included breast cancer, thyroid cancer, and cervical cancer. The prevalence of breast cancer is the fraction (%) of respondents who answered “Yes” to whether they had been diagnosed with breast cancer among those who answered “Yes” in response to whether they had a history of malignant tumor. The prevalence of thyroid cancer is the fraction (%) of respondents who answered “Yes” to whether they were diagnosed with thyroid cancer, among those who answered “Yes” to whether they had a history of malignant tumors. The prevalence of cervical cancer is the fraction (%) of respondents who answered “Yes” to whether they had been diagnosed with cervical cancer, among those who answered “Yes” to whether they had a history of malignant tumors.

*Sociodemographic characteristics*: Each of the sociodemographic variables was classified into three groups: age as 40 years to 49 years, 50 to 59 years, and 60 years or older; education levels as middle school or lower, high school, and higher education; monthly household income into under 2 million Korean won (KRW), 2 million to 4 million KRW, and 4 million KRW or more.

*Health behaviors*: Smoking status was classified as never smoked, former smoker, and current smoker; drinking status as never drinker, former drinker, and current drinker; regular exercise (enough to sweat) as yes or no; BMI as underweight (<18.5 kg/m^2^), normal weight (18.5–22.9 kg/m^2^), overweight (23–24.9 kg/m^2^), and obese (≥25 kg/m^2^).

*Reproductive history*: The age at menarche was classified as under 13 years and 13 years or older; age at menopause into five groups: under 40 years, 40 to 44 years, 45 to 49 years, 50 to 54 years, and 55 years or older. The number of breastfed children was classified into one, two, and three or more; age at first childbirth into three groups: age as under 20 years, 20 to 29 years, 30 to 39 years, and 40 years older.

### Bias

No sampling bias was expected since all targets were included in the analysis.

### Study size

Because all target population was included, no study size estimation was needed.

### Data analysis

The significance level of statistical analysis was set to .05. The chi-square test or Fisher exact test was performed to determine the difference in the prevalence of female cancers according to demographic characteristics, health behaviors, and reproductive history. To determine the association between female cancers and breastfeeding experience, the number of childbirths, and total breastfeeding duration, logistic regression was used. The chi-square test was used to test the model of logistic regression analysis. Four models were constructed for logistic regression analysis to correct for potential confounders: Model 1 (simple odds ratio [OR]), Model 2 (adjustment for sociodemographic characteristics), Model 3 (adjustment for sociodemographic characteristics, and health behaviors), and Model 4 (adjustment for sociodemographic characteristics, health behaviors, and reproductive history). An OR and 95% confidence interval (CI) were calculated. Data were analyzed using SAS ver. 9.4 statistics program (SAS Institute, Cary, NC, USA).

## Results

### Prevalence of female cancers according to sociodemographic characteristics, health behaviors, and reproductive history

Table 1 shows the distribution of total population (N=113, 944) by sociodemographic characteristics, health behaviors, and reproductive history.

Analysis of the difference in the prevalence of female cancers according to sociodemographic characteristics resulted in the verification of significant differences in the prevalence of breast cancer, thyroid cancer, and cervical cancer in the age groups (*p*<.001). More specifically, the prevalence of breast and thyroid cancer was high in women in their 50s, and the prevalence of cervical cancer in women in their 60s or older. In addition, the prevalence of thyroid cancer and cervical cancer showed a significant difference depending on education level and monthly household income; thyroid cancer was highest in the women with access to higher education and monthly household income of 4 million KRW or more, while cervical cancer was highest in the women educated till middle school or lower and a monthly household income of less than 2 million KRW (Table 1).

Analysis of the difference in the prevalence of female cancers according to health behaviors is as follows. Higher prevalence of breast cancer was observed in the never-smoked group (*p*=.001), former drinker group (*p*<.001), regular exercise group (*p*<.001), and the underweight group (*p*=.002). Higher prevalence of thyroid cancer was observed in the former drinker group (*p*<.001) and regular exercise group (*p*<.001). Higher prevalence of cervical cancer was observed in the former smoker group (*p*=.048), former drinker group (*p*=.010), and underweight group (*p*=.013) (Table 1).

Analysis of the difference in the prevalence of female cancers according to reproductive history is as follows. The prevalence of breast cancer showed a significant difference depending on the age at menopause (*p*<.001), the experience of childbirth (*p*=.040), the number of childbirths (*p*<.001), the age at first birth (*p*=.033), and breastfeeding experience (*p*<.001). Higher prevalence of breast cancer was associated with the age of 40 to 44 years at menopause, no experience of childbirth, one childbirth, the age of 40 years or older at first childbirth, and non-breastfeeding. The prevalence of thyroid cancer showed a significant difference depending on the age at menarche (*p*=.041), number of childbirths (*p*=.002), age at first childbirth (*p*=.025), and total breastfeeding duration (*p*<.001). Higher prevalence of thyroid cancer was observed in those who had menarche before the age of 13 years, two childbirths, were aged 30 to 39 years at first childbirth, and total less than 6 months breastfeeding duration. The prevalence of cervical cancer was found to be significantly associated with the age at menarche (*p*=.035), age at menopause (*p*<.001), number of childbirths (*p*<.001), age at first childbirth (*p*<.001), and total breastfeeding duration (*p*=.012). Higher prevalence of cervical cancer was observed in the age of 13 years or older at menarche, the age of under 40 years at menopause, more than three childbirths, the age of under 20 years at first childbirth, and total of more than 36 months breastfeeding duration (Table 1).

### Effect of breastfeeding experience, number of childbirths, and total breastfeeding duration on the prevalence of female cancers

**Figure 1.**
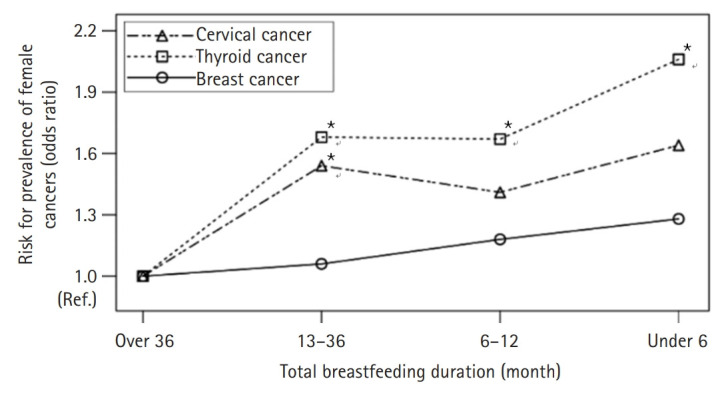
The relationship between total breastfeeding duration and breast, thyroid, and cervical cancer. ^*^p<.05.

First, a simple logistic regression analysis (Model 1) of the effects of breastfeeding experience (Yes/No), number of childbirths, and total breastfeeding duration on the prevalence of breast cancer resulted in the verification that higher breast cancer is associated with non-breastfeeding, lower number of childbirths, and shorter total breastfeeding duration. The OR after adjustment for sociodemographic characteristics, that is, age, education level, and monthly household income (Model 2) was 1.50 (95% CI, 1.23–1.82) for non-breastfeeding compared to breastfeeding; 1.64 (95% CI, 1.36–1.99) for two childbirths versus more than three and 1.89 (95% CI, 1.45–2.47) for one childbirth versus more than three; 1.42 (95% CI, 1.09–1.85) for total breastfeeding duration of 13 to 36 months versus more than 36 months, 1.60 (95% CI, 1.18–2.19) for 6 to 12 months versus more than 36 months, and 1.80 (95% CI, 1.25–2.60) for less than 6 months versus more than 36 months. The OR after additional adjustment for health behaviors (smoking status, drinking status, regular exercise, and BMI) to Model 2 (Model 3) was 1.51 (95% CI, 1.24–1.83) for the non-breastfeeding group; 1.62 (95% CI, 1.34–1.96) and 1.92 (95% CI, 1.47–2.51) for two childbirths and one childbirth, respectively; 1.39 (95% CI, 1.07–1.81), 1.58 (95% CI, 1.16–2.15), and 1.78 (95% CI, 1.24–2.57) for the total breastfeeding duration of 13 to 36 months, 6 to 12 months, and less than 6 months, respectively, versus more than 36 months. However, the OR after additional adjustment for reproductive history (age at menarche, age at menopause, number of breastfed children, age at first childbirth) to Model 3 (Model 4) showed 1.37 (95% CI, 1.09–1.72) higher risk of developing breast cancer in the non-breastfeeding group, and no statistically significant differences were observed in relation to the number of childbirths and the total breastfeeding duration (Table 2, Figure 1).

For thyroid cancer prevalence, a simple logistic regression analysis (Model 1) of the effects of breastfeeding experience, number of childbirths, and total breastfeeding duration on the prevalence of thyroid cancer yielded the results that the number of childbirths and total breastfeeding duration affected the prevalence of thyroid cancer and no statistically significant association was observed between breastfeeding experience and thyroid cancer. The OR after adjustment for sociodemographic characteristics, that is, age, education level, and monthly household income (Model 2) was 1.24 (95% CI, 1.03–1.48) for two childbirths versus more than three; 1.62 (95% CI, 1.23–2.13), 1.49 (95% CI, 1.08–2.05), and 1.48 (95% CI, 1.02–2.14) for the total breastfeeding duration of 13 to 36 months, 6 to 12 months, and less than 6 months, respectively, versus more than 36 months. The OR after additional adjustment for health behaviors (smoking status, drinking status, regular exercise, and BMI) to Model 2 (Model 3) was 1.26 (95% CI, 1.05–1.52) for two childbirths versus more than three; number of childbirths; 1.62 (95% CI, 1.23–2.13), 1.52 (95% CI, 1.10–2.10), and 1.54 (95% CI, 1.06–2.23) for the total breastfeeding duration of 13 to 36 months, 6 to 12 months, and less than 6 months, respectively, versus more than 36 months. The OR after additional adjustment for reproductive history (age at menarche, age at menopause, number of breastfed children, age at first childbirth) to Model 3 (Model 4) showed no statistically significant differences in relation to breastfeeding, but 1.68 (95% CI, 1.25–2.25), 1.67 (95% CI, 1.13–2.48), and 2.06 (95% CI, 1.28–3.31) for the total breastfeeding duration of 13 to 36 months, 6 to 12 months, and less than 6 months, respectively, versus more than 36 months, showing the greatest thyroid cancer risk at the shortest total breastfeeding duration (Table 2, Figure 1).

Lastly, for cervical cancer prevalence, a simple logistic regression analysis (Model 1) of the effects of breastfeeding experience, number of childbirths, and total breastfeeding duration on the prevalence of cervical cancer resulted in the findings that number of childbirths and total breastfeeding duration are factors affecting the prevalence of cervical cancer. However, in Model 2 constructed by adjusting for sociodemographic characteristics (age, education level, and monthly household income) and Model 3 constructed by additionally adjusting for health behaviors (smoking status, drinking status, regular exercise, and BMI) to Model 2, no statistically significant factors were verified. In Model 4 constructed by additionally adjusting for reproductive history (age at menarche, age at menopause, number of breastfed children, and age at first childbirth) to Model 3, the OR was 1.54 (95% CI, 1.12–2.11) for total breastfeeding duration of 13 to 36 months versus more than 36 months (Table 2, Figure 1).

## Discussion

This epidemiological study was conducted to determine the association between breastfeeding and the prevalence of breast, thyroid, and cervical cancers as the major female cancers in Korean women based on the KoGES.

### Higher prevalence of breast cancer in the non-breastfeeding group

Finally, after adjusting sociodemographic characteristics, health behaviors, and reproductive history, the prevalence of breast cancer was 1.37 times higher in the non-breastfeeding group compared with the breastfeeding group. However, no statistically significant differences were observed in the number of childbirths and total breastfeeding duration. It is worth noting that even after adjusting for various sociodemographic and lifestyle factors related to breast cancer risk, as well as reproductive history, which can be considered as the exposure factor to sex hormones, the prevalence of breast cancer was higher in the non-breastfeeding group compared with the breastfeeding group. In the study conducted by Park et al. [20], the relative risk for breast cancer was 1.03 times higher in women with no breastfeeding experience, similar to the result of this study. It is noteworthy that breastfeeding’s effect on breast cancer was confirmed even after considering menarche, menopause, childbirth age, and healthy lifestyle, which are reported as major risk factors for breast cancer [20]. However, the result for breastfeeding duration and breast cancer prevalence differs from previous studies. In a non-Korean meta-analysis study, breastfeeding for 12 months or longer reduced the risk of breast cancer compared to non-breastfeeding [14]. Also, in the study conducted by Kim et al. [16], the average duration of breastfeeding of 11 to 12 months reduced risk of breast cancer by 54% compared with the duration of 1 to 4 months among Korean women. The variable about breastfeeding duration is a secondary data collected in a self-reported form for total breastfeeding period, which should be considered as it has a limitation to the precision of the data analysis. There is a need to analyze the risk of breast cancer depending on the breastfeeding duration in Korean women through the prospective research design.

### Lower prevalence of thyroid cancer in the longer breastfeeding duration group

After adjusting for the sociodemographic characteristics, health behavioral factors recommended for cancer prevention [6], and reproductive history in Korean adult women (40 years or older), the prevalence of thyroid cancer was 1.68, 1.67, and 2.06 times higher for the total breastfeeding duration of 13 to 36 months, 6 to 12 months, and less than 6 months, respectively, versus more than 36 months with statistical significance. This is similar to the results of a non-Korean study in which it was verified that longer breastfeeding duration was associated with a lower prevalence of thyroid cancer [15]. The study of some female thyroid cancer patients in Korea also reported that breastfeeding period and the number of breastfeeding babies were associated with the risk of thyroid cancer [18]. A dose-response relationship between the total breastfeeding duration and the prevalence of thyroid cancer has thus been potentially confirmed in Korean women as well. Although it could not explain the biological mechanism underlying the inverse association between breastfeeding and thyroid cancer fully, it was understood that breastfeeding inhibits ovulation and can thus decrease the endogenous estrogen that could affect the proliferation of malignant thyroid cells [15]. A non-Korean study verified the association between female estrogen exposure and thyroid cancer by demonstrating the growth-stimulating effect of estradiol on benign and malignant thyroid cells [21]. The risk of thyroid cancer was reported to decrease in the cases of breastfeeding, oral contraceptive use, and old age at first pregnancy [22]. Along with previous studies, our results support the evidence that breastfeeding may reduce the risk of thyroid cancer in women by suppressing ovulation and reducing exposure to endogenous estrogen. But other reported risk factors that include diabetes, obesity, genetic predisposition, radiation, pollutants, and diet need to be considered [23].

### Increased prevalence of cervical cancer for the shorter breastfeeding duration group

After adjusting for sociodemographic characteristics, health behaviors, and reproductive history, the prevalence of cervical cancer significantly increased by 1.54 times for the total breastfeeding duration of 13 to 36 months versus ≥36 months. There is a lack of previous research on the association between cervical cancer and breastfeeding duration, although a Moroccan study [24] reported that no significant difference was observed in the relationship between breastfeeding and cervical cancer. Therefore, repeat studies are needed to confirm the difference in the incidence rate of cervical cancer depending on breastfeeding experience and breastfeeding duration. The uterine cervix is a part of the female reproductive tract that responds very well to estrogen [25]. A recent meta-analysis reported that breastfeeding reduces the risk of estrogen-affected endometrial cancer, which suggests the need to examine the relationship between cervical cancer and breastfeeding in the given context [26]. And it was reported that contraceptives, obstetric history, sexually transmitted diseases, tobacco, nutrition, and immune system are factors that affect the development of cervical cancer [10,27]. When epidemiologists study the relationship between breastfeeding and cervical cancer in the future, other risk factors, including HPV infection, which is known to be the main etiology of cervical cancer, should be considered.

This study has the limitation of not being able to clarify the causal relation between breastfeeding and female cancers and the nature of secondary data analysis limiting including other specific variables. We suggest future research to conduct repeat studies in a large-scale cohort study and case-control designs to elucidate the epidemiological relationship and mechanisms that can link breastfeeding and the occurrence of female cancer.

In conclusion, this study was conducted to identify the association between breastfeeding and the prevalence of breast, thyroid, and cervical cancer as the leading female cancers, based on the representative KoGES urban cohort. Even after adjusting for sociodemographic characteristics, health behavior, and reproductive history, the prevalence of breast cancer was higher in the non-breastfeeding group, and the prevalence of thyroid and cervical cancer became higher with a decrease in the total breastfeeding duration.Future studies are also needed on modifiable factor, there may be various factors related to female cancers, such as stress, environment, and lifestyle. Currently, in Korea, the rate of exclusive breastfeeding for infants under 6 months of age is as low as 18.3%, which is far below the 50% target of the World Health Organization [28]. Over the past 20 years, exclusive breastfeeding rate in developed countries has increased sharply [29] and 40% of infants under 6 months of age are exclusively breastfed globally [11]. Thus is necessary to double efforts to raise the breastfeeding compliance rate of Korean women. Results of this study are significant in that they can be used as the basic data for establishing the rationale for setting up policy promotional efforts to encourage breastfeeding compliance for the prevention of female cancer among Korean women.

## Figures and Tables

**Table 1.** Prevalence of female cancers according to sociodemographic characteristics, health behaviors, and reproductive history (N=113,944)

Variable	Categories	Total population, N (%)	Prevalence of breast cancer, n (%)	χ^2^ test (*p*)	Prevalence of thyroid cancer, n (%)	χ^2^ t-test (*p*)	Prevalence of cervical cancer, n (%)	χ^2^ test (*p*)
*Sociodemographic characteristics*								
Age (year)				36.95 (<.001)		16.60 (<.001)		82.88 (.001)
	40–49	43,460 (38.1)	263 (0.6)		291 (0.7)		163 (0.4)	
	50–59	45,418 (39.9)	437 (1.0)		404 (0.9)		248 (0.6)	
	≥60	25,066 (22.0)	217 (0.9)		172 (0.7)		229 (0.9)	
Education level				4.04 (.133)		22.73 (<.001)		19.06 (.001)
	≤Middle school	44,362 (39.7)	333 (0.8)		273 (0.6)		302 (0.7)	
	High school	41,997 (37.5)	344 (0.8)		363 (0.9)		217 (0.5)	
	>High school	25,499 (22.8)	227 (0.9)		224 (0.9)		113 (0.4)	
Monthly household income (KRW)								
	<2 million	32,705 (35.1)	300 (0.9)	2.78 (.249)	233 (0.7)	12.31 (.002)	248 (0.8)	34.92 (<.001)
	2–4 million	39,064 (41.9)	317 (0.8)		352 (0.9)		206 (0.5)	
	>4 million	21,509 (23.0)	175 (0.8)		209 (1.0)		82 (0.4)	
*Health behaviors*								
Smoking status							
	No (never)	109,782 (96.4)	905 (0.8)	15.39 (.001)	846 (0.8)	4.07 (.131)	605 (0.6)	6.07 (.048)
	Yes (former)	1,489 (1.3)	7 (0.5)		9 (0.6)		13 (0.9)	
	Yes (current)	2,673 (2.3)	5 (0.2)		129 (0.5)		22 (0.8)	
Drinking status								
	No (never)	75,260 (66.7)	730 (1.0)	146.78 (<.001)	629 (0.8)	21.94 (<.001)	452 (0.6)	9.28 (.010)
	Yes (former)	2,482 (2.2)	50 (2.0)		26 (1.1)		20 (0.8)	
	Yes (current)	35,141 (31.1)	136 (0.4)		207 (0.6)		167 (0.5)	
Regular exercise								
	Yes	57,072 (50.5)	558 (1.0)	40.66 (<.001)	509 (0.9)	23.99 (<.001)	315 (0.6)	0.32 (.579)
	No	55,972 (49.5)	357 (0.6)		357 (0.6)		323 (0.6)	
	Body mass index							
	Underweight	2,825 (2.5)	37 (1.3)	15.19 (.002)	17 (0.6)	7.28 (.064)	22 (0.8)	10.71 (.013)
	Normal	47,586 (41.8)	406 (0.9)		347 (0.7)		231 (0.5)	
	Overweight	30,288 (26.6)	242 (0.8)		264 (0.9)		176 (0.6)	
	Obesity	33,245 (29.1)	232 (0.7)		239 (0.7)		211 (0.6)	
*Reproductive history*								
Age at menarche (year)								
	<13	5,130 (4.7)	48 (0.9)	0.91 (.340)	53 (1.0)	4.56 (.041)	18 (0.4)	4.41 (.035)
	≥13	104,697 (95.3)	851 (0.8)		801 (0.8)		603 (0.6)	
Age at menopause (year)								
	<40	3,150 (5.1)	46 (1.5)	124.81 (<.001)	34 (1.1)	2.23 (.693)	113 (3.6)	471.33 (<.001)
	40–44	5,409 (8.7)	130 (2.4)		49 (0.9)		117 (2.2)	
	45–49	18,892 (30.5)	279 (1.5)		168 (0.9)		180 (1.0)	
	50–54	28,642 (46.3)	239 (0.8)		239 (0.8)		98 (0.3)	
	≥55	5,833 (9.4)	42 (0.7)		53 (0.9)		34 (0.6)	
Parity								
	Yes	108,175 (98.8)	852 (0.8)	4.48 (.040)	827 (0.8)	0.00 (.873)	615 (0.6)	0.25 (.851)
	No	1,295 (1.2)	17 (1.3)		10 (0.8)		6 (0.5)	
Number of childbirths (person)								
	1	11,002 (10.2)	110 (1.0)	22.11 (<.001)	88 (0.8)	12.24 (.002)	47 (0.4)	22.03 (<.001)
	2	61,200 (56.8)	519 (0.9)		513 (0.8)		310 (0.5)	
	≥3	35,537 (33.0)	220 (0.6)		223 (0.6)		255 (0.7)	
Age at first childbirth (year)								
	<20	1,762 (1.6)	7 (0.4)	8.77 (.033)	5 (0.3)	9.32 (.025)	21 (1.2)	26.04 (<.001)
	20–29	93,808 (87.6)	727 (0.8)		721 (0.8)		551 (0.6)	
	30–39	11,237 (10.5)	109 (1.0)		99 (0.9)		35 (0.3)	
	≥40	266 (0.3)	3 (1.1)		0(0.0)		1 (0.4)	
Breastfeeding experience								
	Yes	93,346 (86.5)	700 (0.8)	13.62 (<.001)	712 (0.8)	0.10 (.759)	546 (0.6)	2.83 (.098)
	No	14,609 (13.5)	152 (1.0)		115 (0.8)		69 (0.5)	
Total breastfeeding duration (month)								
	<6	9,379 (10.3)	78 (0.8)	6.94 (.074)	80 (0.9)	17.96 (<.001)	46 (0.5)	10.98 (.012)
	6–12	18,668 (20.5)	147 (0.8)		152 (0.8)		94 (0.5)	
	13–36	47,348 (51.9)	373 (0.8)		395 (0.8)		271 (0.6)	
	>36	15,770 (17.3)	94 (0.6)		80 (0.5)		118 (0.8)	

KRW: Korean won (1 million KRW is approximately 900 US dollars).

**Table 2.** Breastfeeding experience, number of childbirths and total breastfeeding duration, and the association of female cancers (breast cancer, thyroid cancer, and cervical cancer) (N=113,944)

Variable	Categories	Number of total population	Number of cancer patients	OR (95% CI)			
				Model 1	Model 2	Model 3	Model 4
*Breast cancer*							
Breastfeeding experience							
	Yes	93,346	700	1.0	1.0	1.0	1.0
	No	14,609	152	1.39 (1.17–1.66)^*^	1.50 (1.23–1.82)^*^	1.51 (1.24–1.83)^*^	1.37 (1.09–1.72)^*^
Number of childbirths (times)							
	≥3	35,537	220	1.0	1.0	1.0	1.0
	2	61,200	519	1.37 (1.17–1.61)^*^	1.64 (1.36–1.99)^*^	1.62 (1.34–1.96)^*^	1.38 (0.87–2.19)
	1	11,002	110	1.62 (1.29–2.04)^*^	1.89 (1.45–2.47)^*^	1.92 (1.47–2.51)^*^	0.97 (0.54–1.75)
Total breastfeeding duration (month)							
	>36	15,770	94	1.0	1.0	1.0	1.0
	13–36	47,348	373	1.32 (1.06–1.66)^*^	1.42 (1.09–1.85)^*^	1.39 (1.07–1.81)^*^	1.06 (0.79–1.42)
	6–12	18,668	147	1.32 (1.02–1.72)^*^	1.60 (1.18–2.19)^*^	1.58 (1.16–2.15)^*^	1.18 (0.81–1.72)
	<6	9,379	78	1.40 (1.03–1.89)^*^	1.80 (1.25–2.60)^*^	1.78 (1.24–2.57)^*^	1.28 (0.81–2.03)
*Thyroid cancer*							
Breastfeeding experience							
	Yes	93,346	712	1.0	1.0	1.0	1.0
	No	14,609	115	1.03 (0.85–1.26)	0.96 (0.78–1.19)	0.98 (0.80–1.21)	0.94 (0.69–1.27)
Number of childbirths (person)							
	≥3	35,537	226	1.0	1.0	1.0	1.0
	2	61,200	513	1.32 (1.13–1.55)^*^	1.24 (1.03–1.48)^*^	1.26 (1.05–1.52)^*^	1.04 (0.67–1.62)
	1	11,002	88	1.26 (0.98–1.61)	1.20 (0.91–1.57)	1.26 (0.96–1.65)	1.28 (0.69–2.38)
Total breastfeeding duration (month)							
	>36	15,770	80	1.0	1.0	1.0	1.0
	13–36	47,348	395	1.65 (1.30–2.10)^*^	1.62 (1.23–2.13)^*^	1.62 (1.23–2.13)^*^	1.68 (1.25–2.25)^*^
	6–12	18,668	152	1.61 (1.23–2.11)^*^	1.49 (1.08–2.05)^*^	1.52 (1.10–2.10)^*^	1.67 (1.13–2.48)^*^
	<6	9,379	80	1.69 (1.24–2.30)^*^	1.48 (1.02–2.14)^*^	1.54 (1.06–2.23)^*^	2.06 (1.28–3.31)^*^
*Cervical cancer*							
Breastfeeding experience							
	Yes	93,346	546	1.0	1.0	1.0	1.0
	No	14,609	69	0.81 (0.63–1.04)	0.99 (0.75–1.31)	0.98 (0.74–1.29)	0.95 (0.69–1.30)
Number of childbirths (person)							
	≥3	35,537	255	1.0	1.0	1.0	1.0
	2	61,200	310	0.70 (0.60–0.83)	0.97 (0.79–1.19)	0.97 (0.51–1.06)	0.85 (0.53–1.36)
	1	11,002	47	0.59 (0.60–0.81)	0.76 (0.53–1.10)	0.74 (0.51–1.06)	0.76 (0.39–1.51)
Total breastfeeding duration (month)							
	>36	15,770	118	1.0	1.0	1.0	1.0
	13–36	47,348	271	0.76 (0.62–0.95)	1.01 (0.78–1.30)	1.01 (0.78–1.31)	1.54 (1.12–2.11)^*^
	6–12	18,668	94	0.67 (0.51–0.88)	1.00 (0.71–1.40)	1.00 (0.71–1.40)	1.41 (0.92–2.15)
	<6	9,379	46	0.65 (0.47–0.92)	1.20 (0.79–1.81)	1.19 (0.79–1.81)	1.64 (0.98–2.75)

Model 1: Unadjusted. Model 2: adjusted for age (40–49, 50–59, ≥60 years), education level (≤middle school, high school, >high school), monthly household income (<2 million Korean won [KRW], 2 million–4 million KRW, >4 million KRW). Model 3: adjusted for Model 2 and smoking status (never, former smoker, current smoker), drinking status (never, former drinker, current drinker), regular exercise (yes or no), body mass index (underweight, normal, overweight, obesity). Model 4: adjusted for Model 3 and age at menarche (<13, ≥13 years), age at menopause (<40, 40–44, 45–49, 50–54, ≥55 years), number of breastfed children (1, 2, ≥3 persons), age at first childbirth (<20, 20–29, 30–39, ≥40 years).

* *p*<.05.
